# Supplementary material for: Selective control of prefrontal neural timescales by parietal cortex
Source: Nat Commun. 2026 Mar 9;17:3687. doi: 10.1038/s41467-026-70326-1 (PMC13100148; doi:10.1038/s41467-026-70326-1)
Supplement: Supplementary file 1 — Supplementary Information [file 41467_2026_70326_MOESM1_ESM.pdf]

Supplementary information

Figures

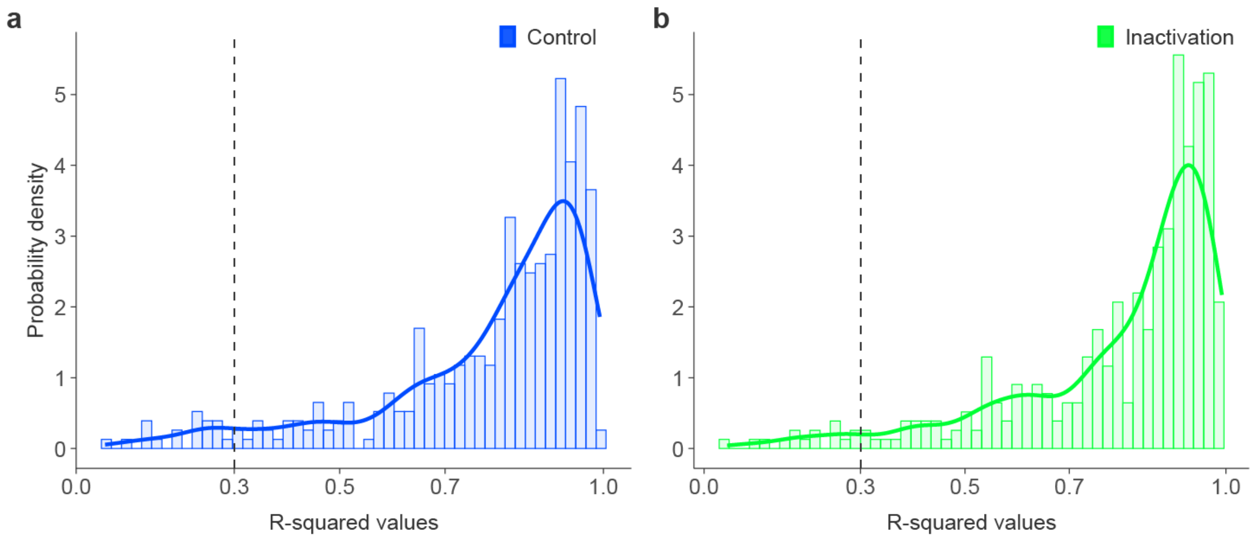

Fig. S1 | Distribution of R^2 values from exponential decay fittings, related to Fig. 1.

(a) Histogram showing the distribution of R^2 values obtained from fitting an exponential decay function to the baseline spike-count autocorrelation of each FEF neuron in the control condition (blue; $n = 380$ neurons). (b) Same as (a), but for the PPC inactivation condition (green; $n = 372$ neurons). Source data are provided as a Source Data file.

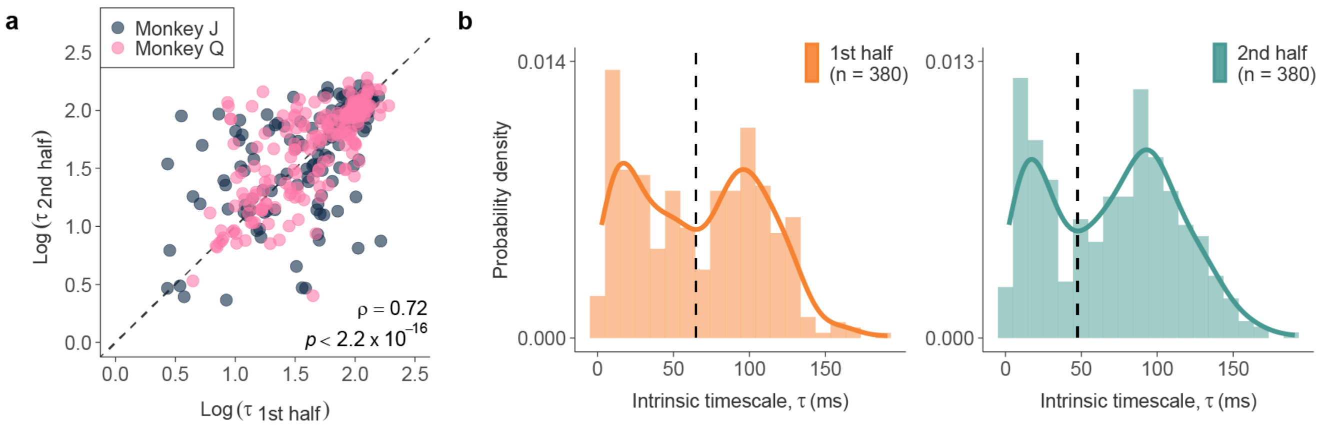

Fig. S2 | Stability of intrinsic neural timescale measurements, related to Fig. 2.

(a) Correlation between intrinsic timescales calculated from the first and second halves of each recording session. Each point represents an FEF neuron ($n = 380$), with color indicating the monkey (Monkey J, $n = 207$; Monkey Q, $n = 173$). Spearman's rank correlation coefficients (ρ) and corresponding p values are indicated in the panel. (b) Histograms and kernel density estimates showing the distribution of intrinsic timescales for the first (left) and second (right) halves of the sessions. Dashed vertical lines indicate the global minimum of the kernel density estimate. Source data are provided as a Source Data file.

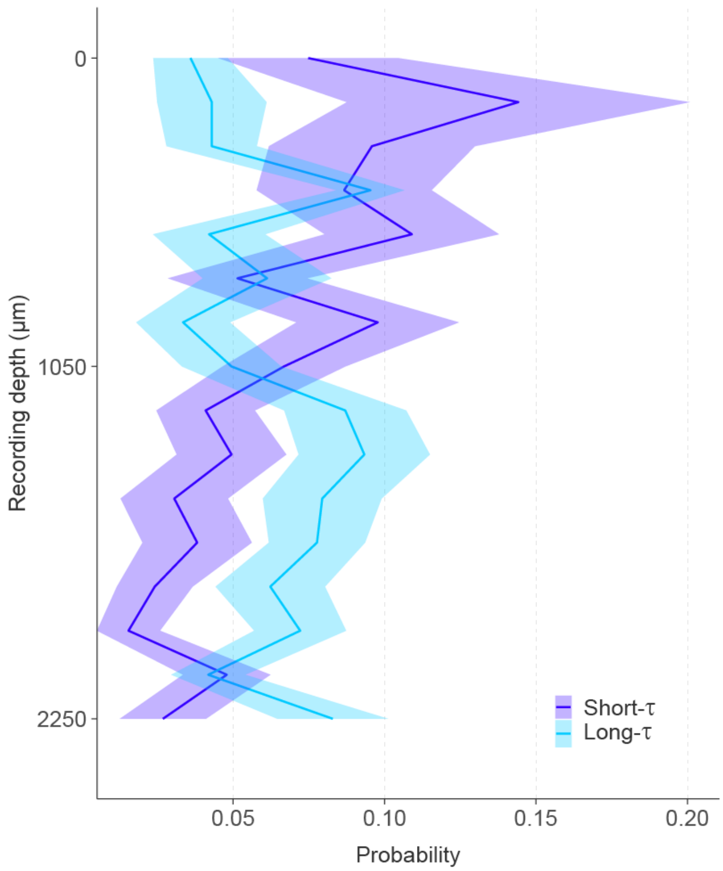

Fig. S3 | Laminar distribution of short- and long-timescale neurons, related to Fig. 2E.

Probability distribution of short-τ (purple) and long-τ (blue) neurons as a function of cortical recording depth (μm). Solid lines show the mean probability across recording sessions ($n = 12$ sessions), and the surrounding shaded regions indicate mean $\pm$ SEM. Source data are provided as a Source Data file.

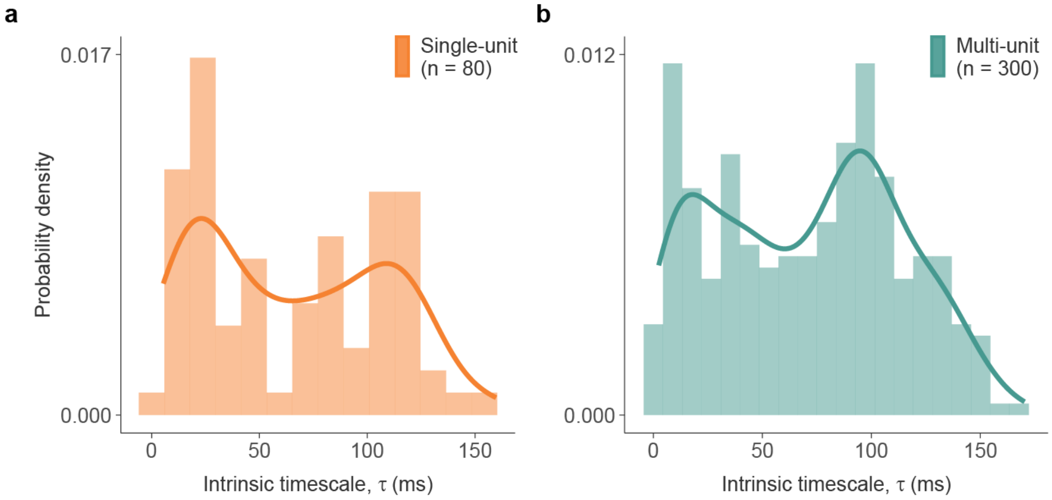

Fig. S4 | Timescale distributions for single- and multi-units, related to Fig. 2.

Histograms and kernel density estimates of intrinsic neural timescales shown separately for single-unit (SUA) and multi-unit (MUA) FEF populations. (a) Distribution for single units (SUA; $n = 80$ neurons; 40 short-τ, 40 long-τ), plotted with 14 histogram bins. (b) Distribution for multi-units (MUA; $n = 300$ units; 128 short-τ, 172 long-τ), plotted with 20 histogram bins. Kernel density estimates used R's default bandwidth ($h = 16.00$ ms for SUA; $h = 12.29$ ms for MUA). Source data are provided as a Source Data file.

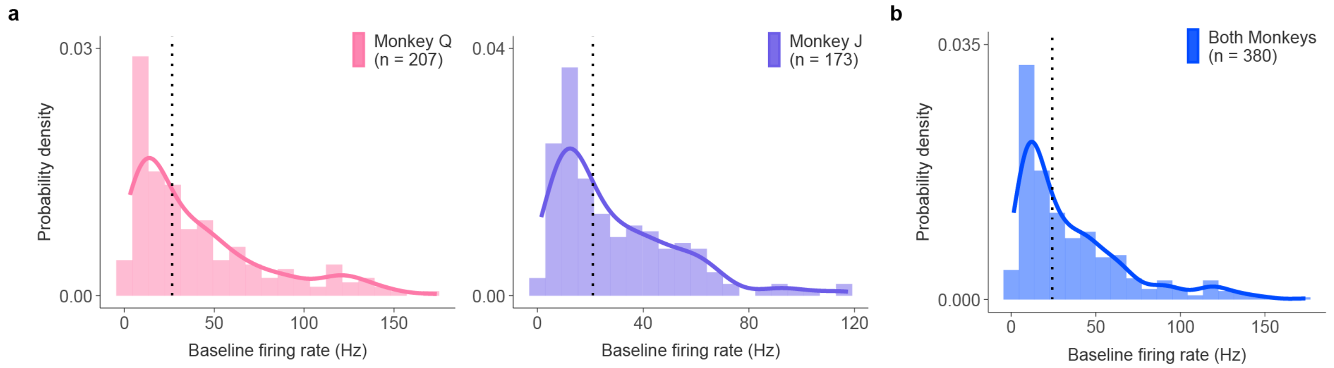

Fig. S5 | Distribution of baseline firing rates in FEF neurons, related to Fig. 2.

(a) Histograms and kernel density estimates of baseline firing rates for Monkey Q (left; $n = 207$ neurons) and Monkey J (right; $n = 173$ neurons). (b) Histogram and kernel density estimate of baseline firing rates for both monkeys combined ($n = 380$). In all panels, solid lines represent the kernel density estimates and dotted vertical lines denote the median firing rate for the respective population. Source data are provided as a Source Data file.

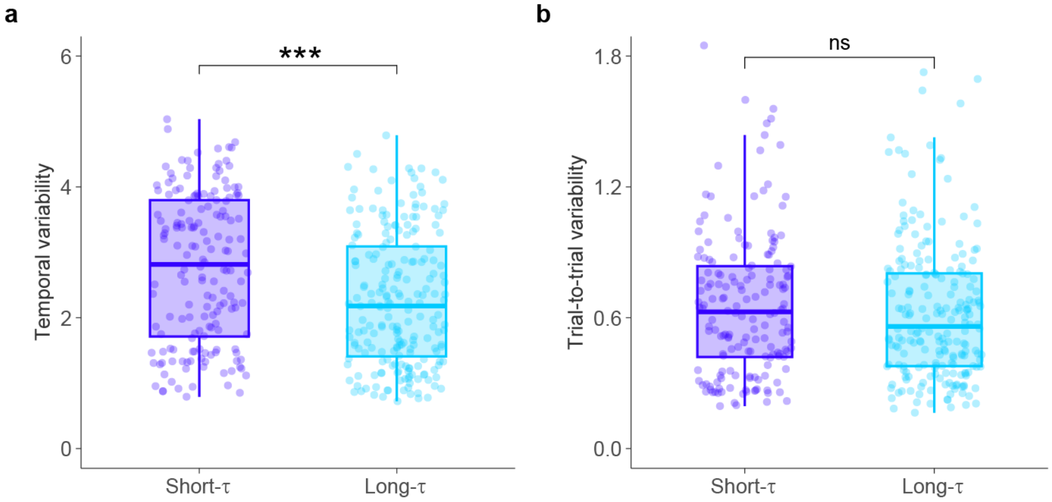

Fig. S6 | Firing variability of short-τ and long-τ neurons, related to Fig. 2.

(a) Comparison of temporal variability in firing rate during the baseline period between short-τ (purple; $n = 168$) and long-τ (blue; $n = 212$) FEF neurons, quantified using the coefficient of variation. (b) Same as (a), but for trial-to-trial variability. Boxplots indicate the median (center line) and interquartile range (box; 25th–75th percentiles), with whiskers extending to 1.5$\times$ the interquartile range. Group differences between short-τ and long-τ neurons were assessed with two-sided Wilcoxon rank-sum tests. Source data are provided as a Source Data file. (***, $p < 0.001$; ns = not significant).

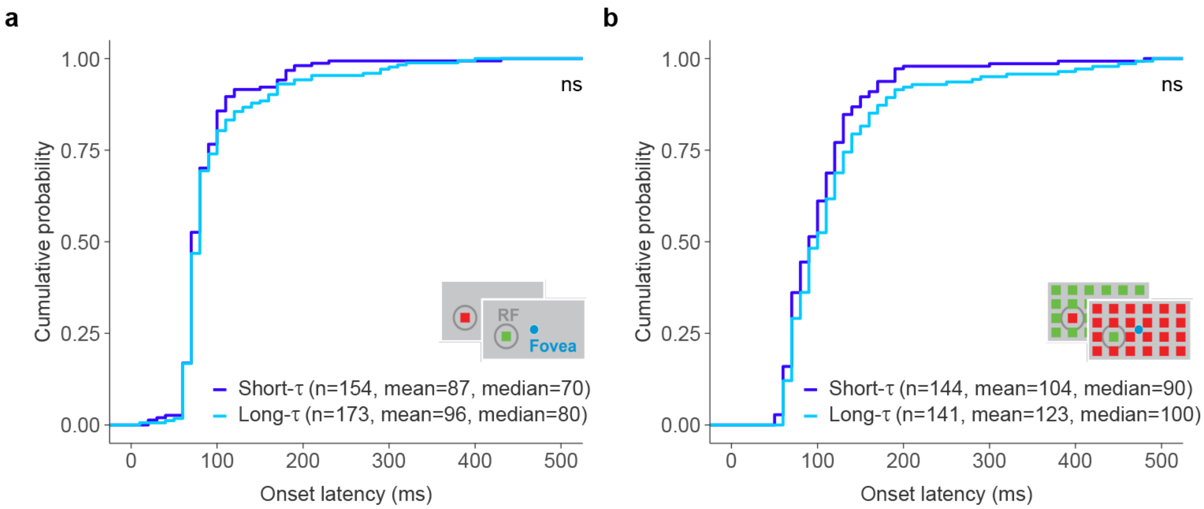

Fig. S7 | Cumulative distributions of visual onset latencies, related to Fig. 3B.

Cumulative distributions of onset latencies (ms) for short-τ (purple) and long-τ (blue) FEF neurons during single-stimulus (a) and popout (b) trials. In (a), the results are based on neurons with a detectable onset in single-stimulus trials (short-τ: $n = 154$; long-τ: $n = 173$). In (b), the results are based on neurons with a detectable onset in popout trials (short-τ: $n = 145$; long-τ: $n = 142$). Differences between short-τ and long-τ distributions were assessed with Cramér–von Mises tests (ns, not significant). Source data are provided as a Source Data file.

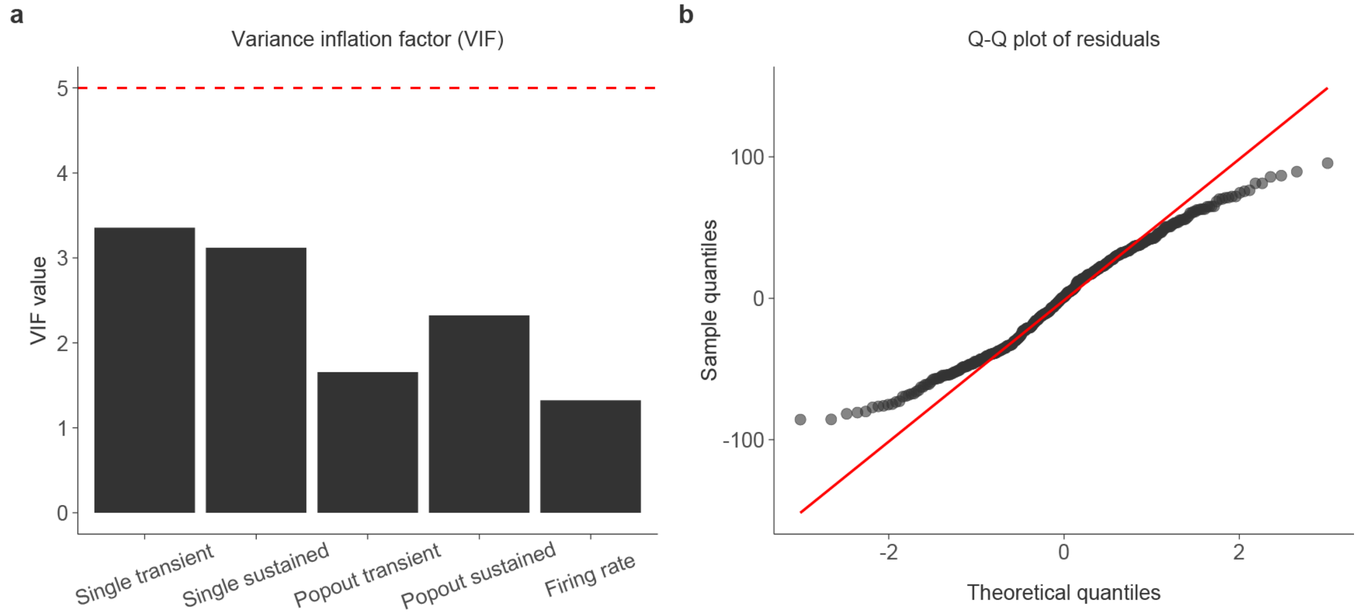

Fig. S8 | Diagnostics for the multiple regression model, related to Fig. 3A.

(a) Variance inflation factor (VIF) plot showing the VIF for each predictor. The red line indicates potential multicollinearity for VIF values exceeding 5. (b) Quantile–Quantile (Q–Q) plot examining the normality of model residuals. Departures from the red reference line suggest deviations from a normal distribution. Source data are provided as a Source Data file.

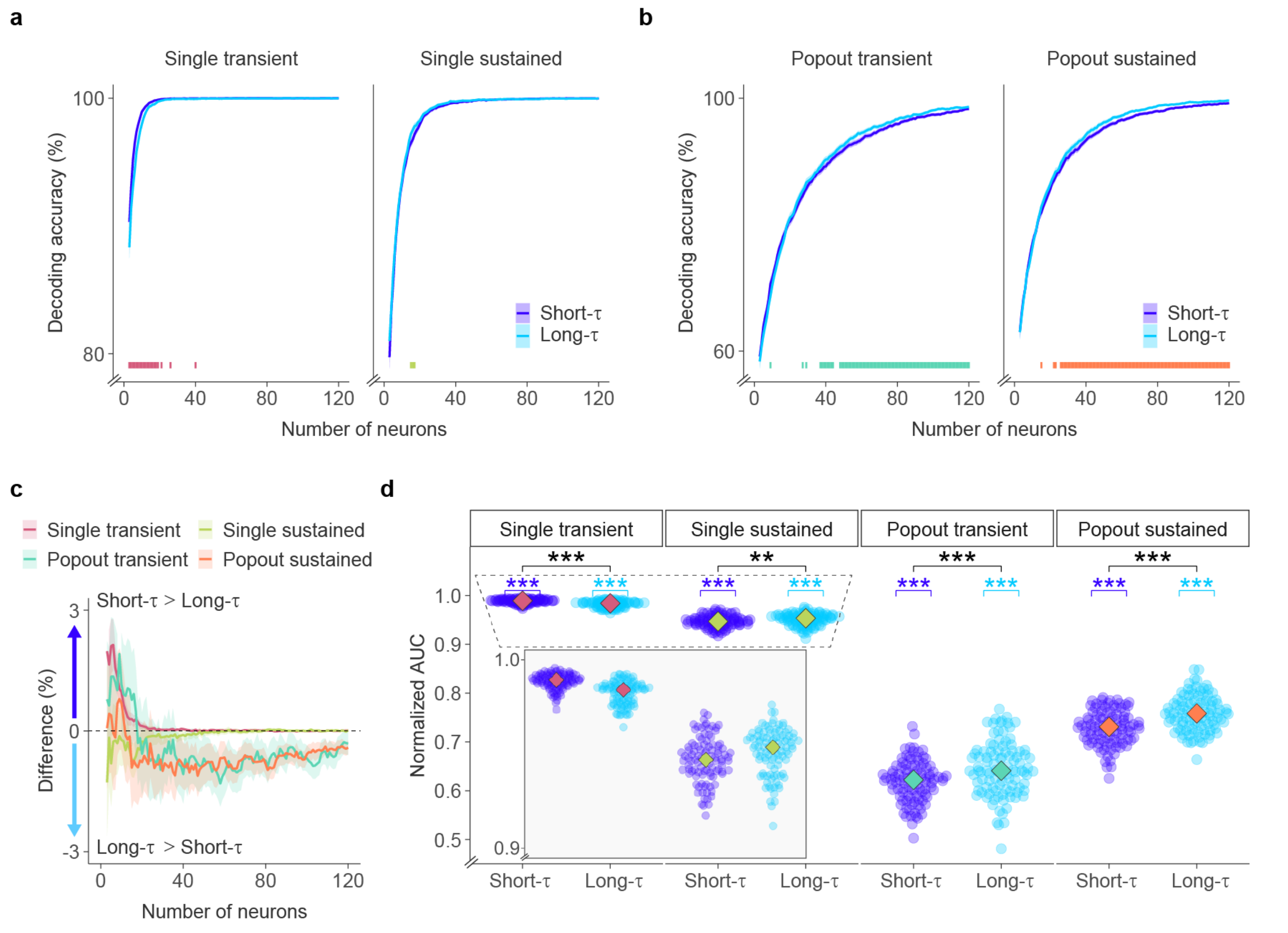

Fig. S9 | Population decoding for stimulus detectability, related to Fig. 3.

(a) Population decoding accuracy for detecting whether a single stimulus was inside versus outside the receptive field, plotted as a function of the number of neurons. Curves show the mean accuracy for short-τ (purple) and long-τ (blue) neuronal populations across 500 decoding iterations. Shaded areas indicate 95% CIs across iterations. Tick marks at the bottom indicate neuron counts where decoding accuracy differed significantly between the two groups (two-sided Wilcoxon rank-sum tests across iterations, $p < 0.05$, FDR-corrected for multiple comparisons). (b) Population decoding accuracy for detecting a popout stimulus, formatted as in (a). (c) Difference in decoding accuracy (short-τ minus long-τ) between the two neuronal populations for each of the four experimental conditions shown in (a) and (b). Shaded areas represent 95% CIs for the differences across iterations. (d) Comparison of normalized area under the curve (AUC) values from the decoding curves in panels (a) and (b) for short-τ and long-τ neurons. Normalized AUC values range from 0 (chance performance) to 1 (perfect decoding across all population sizes). Diamonds indicate medians. Within each condition, normalized AUC values for short-τ and long-τ neurons were tested against zero using two-sided Wilcoxon signed-rank tests, and differences between the two groups were assessed with two-sided Wilcoxon rank-sum tests. Source data are provided as a Source Data file. (**, $p < 0.01$; ***, $p < 0.001$).

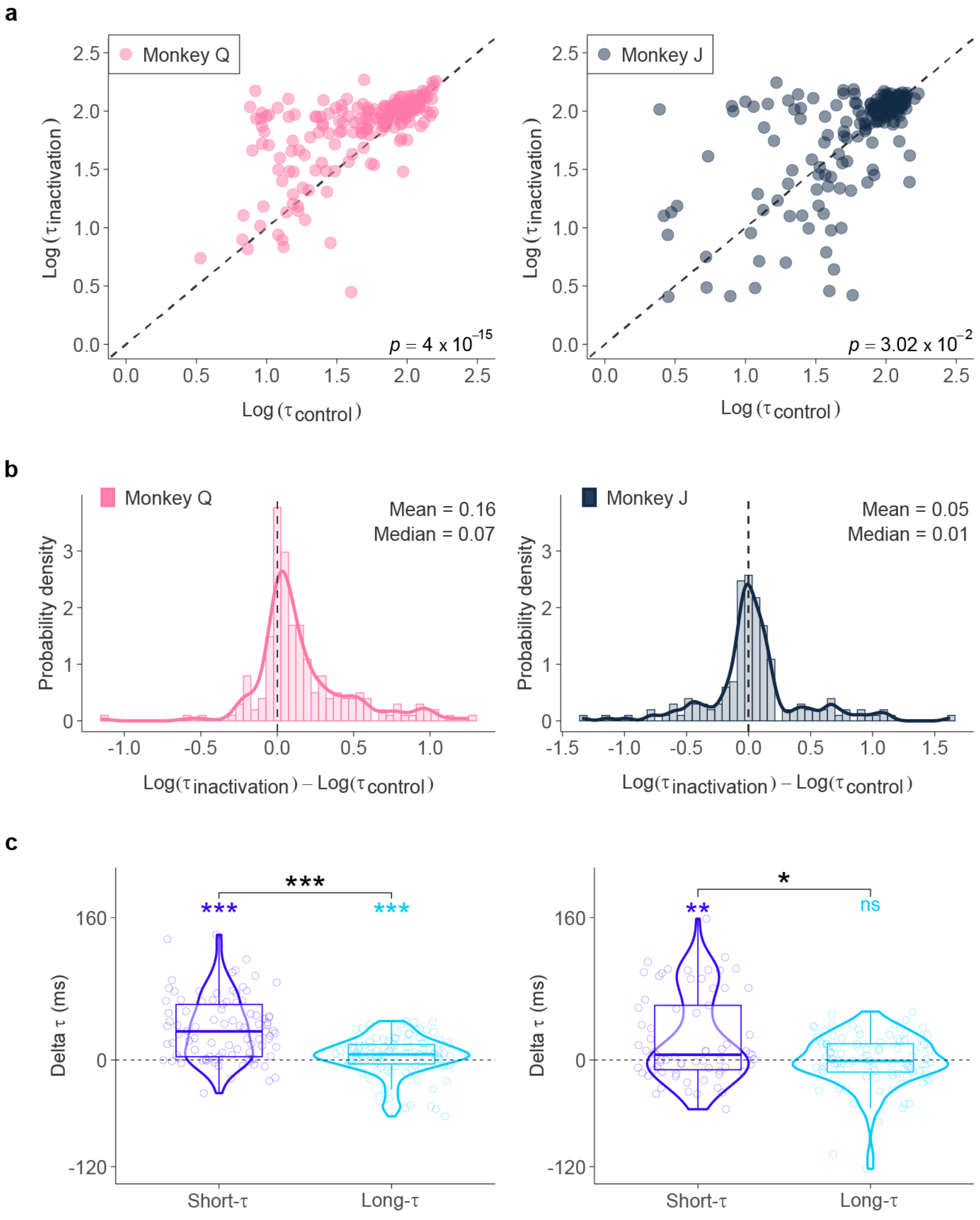

Fig. S10 | PPC inactivation increases intrinsic neural timescales, related to Fig. 4B.

(a) Scatter plots comparing log-transformed intrinsic neural timescales (τ) between the control and PPC inactivation conditions for Monkey Q (left, $n = 205$) and Monkey J (right, $n = 167$). Each point represents an FEF neuron recorded in both conditions. Points above the diagonal line indicate an increase in τ during inactivation. Differences between control and inactivation conditions were assessed with a two-sided Wilcoxon signed-rank test. (b) Histograms showing the distribution of the difference in log-transformed τ (inactivation minus control) for each monkey, based on the same neurons as in (a). (c) Violin plots showing changes in τ values for short-τ (purple) and long-τ (blue) FEF neurons in Monkey Q (left; $n = 93$ for short-τ and $n = 112$ for long-τ) and Monkey J (right; $n = 69$ for short-τ and $n = 98$ for long-τ). Violin shapes show the distribution of $\Delta\tau$; overlaid boxplots indicate the median (center line) and interquartile range (box; 25th–75th percentiles), with whiskers extending to 1.5$\times$ the interquartile range. Each point represents one FEF neuron in the corresponding group and monkey. For each monkey and timescale group, changes in τ were tested against zero using two-sided Wilcoxon signed-rank tests, and differences

between short-τ and long-τ neurons were assessed with two-sided Wilcoxon rank-sum tests. Source data are provided as a Source Data file. (*, $p < 0.05$; **, $p < 0.01$; ***, $p < 0.001$; ns = not significant).

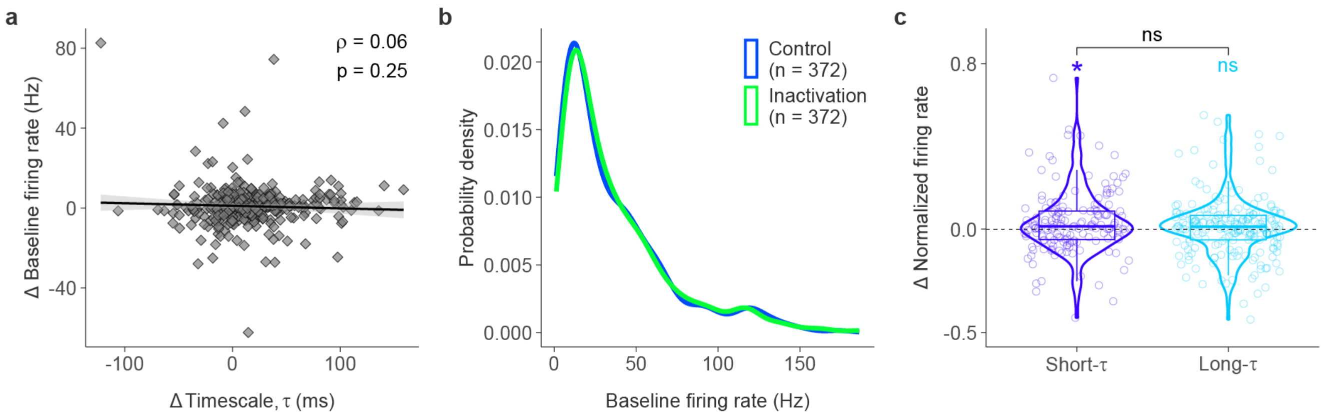

Fig. S11 | Baseline firing rate and its relationship with intrinsic neural timescales following PPC inactivation, related to Fig. 4.

(a) Correlation between changes (Δ) in intrinsic timescales (τ) and baseline firing rates across FEF neurons. Each point represents an FEF neuron ($n = 372$). The association was quantified using a Spearman rank correlation ($\rho = 0.25$, $p = 0.06$). (b) Probability density plots showing the distributions of baseline firing rates across FEF neurons during control (blue) and PPC inactivation (green) conditions ($n = 372$). (c) Violin plots showing normalized changes in baseline firing rates for short-timescale (short-τ, purple) and long-timescale (long-τ, blue) FEF neurons. Violin shapes show the distribution; overlaid boxplots indicate the median (center line) and interquartile range (box; 25th–75th percentiles), with whiskers extending to 1.5$\times$ the interquartile range. Each point represents one neuron (short-τ: $n = 162$; long-τ: $n = 210$). Within each group, changes in firing rate were tested against zero using two-sided Wilcoxon signed-rank tests, and differences between short-τ and long-τ neurons were assessed with a two-sided Wilcoxon rank-sum test. Source data are provided as a Source Data file.

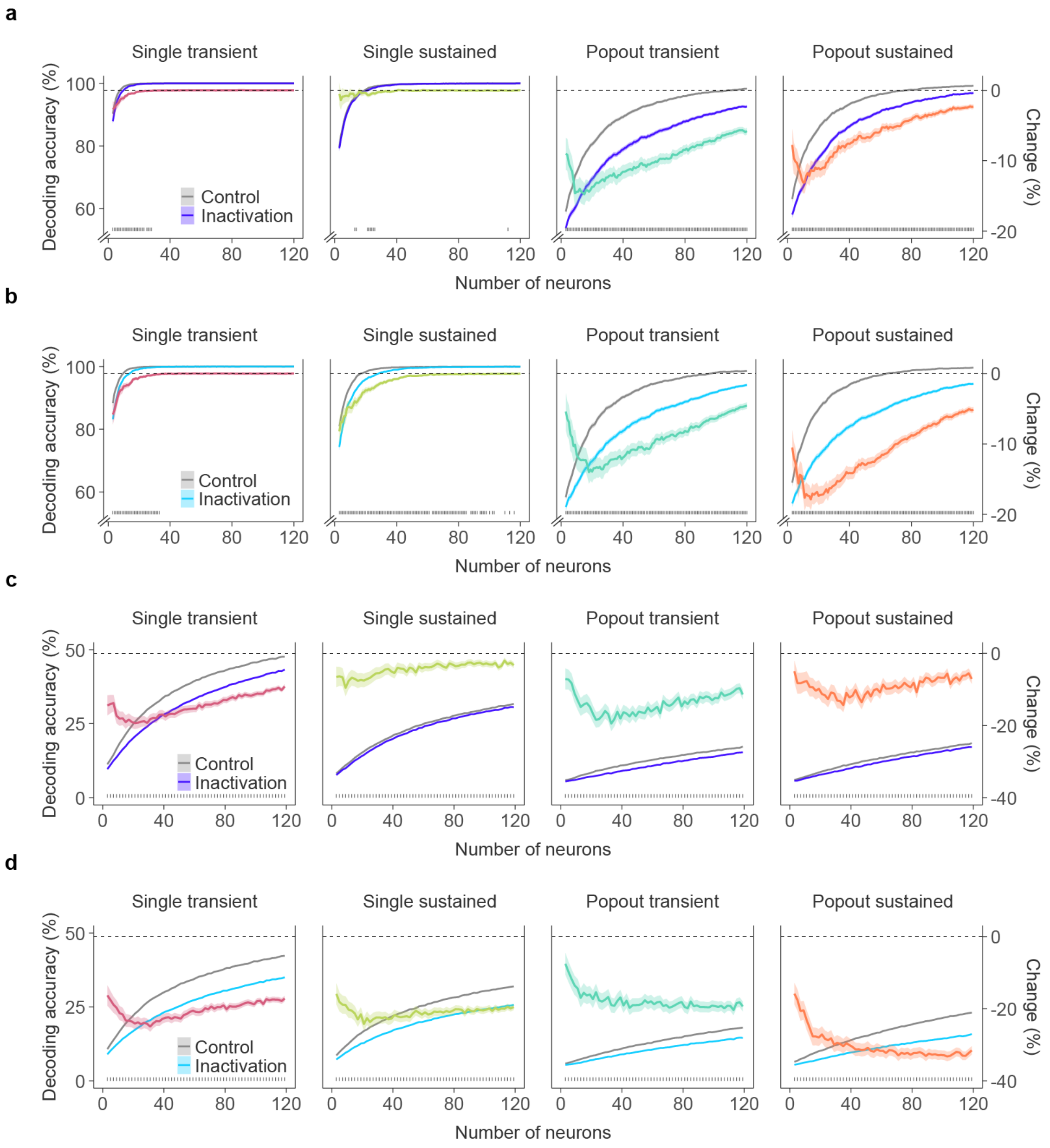

Fig. S12 | Population decoding for stimulus detectability and discriminability during PPC inactivation, related to Fig. 5C–D.

(a) Population decoding performance for the detectability task (inside versus outside the receptive field) in short-τ FEF neurons. The main curves (left y-axis) show decoding accuracy for control (gray) and PPC inactivation (purple) trials as a function of the number of neurons. Shaded areas denote 95% CIs across 500 decoding iterations. The colored curves (right y-axis) show the relative change in accuracy. The horizontal dashed line at zero represents no change. Gray tick marks at the bottom indicate neuron counts with a statistically significant difference between control and inactivation (two-sided Wilcoxon signed-rank tests across iterations, $p < 0.05$, FDR-corrected for multiple comparisons). (b) Same as (a), but for long-τ neurons (inactivation condition shown in blue). (c) Population decoding performance for the discriminability task (decoding the specific stimulus location out of 24 possibilities) in short-τ neurons, formatted as in (a). (d) Same as (c), but for long-τ neurons (inactivation condition shown in blue). Source data are provided as a Source Data file.

Tables

Method	Reference	Monkey(s)	Test statistic	P value
Cramer-von Mises Test	Fisher & Marron, 2001	Both monkeys	0.48	$p < 2.2 \times 10^{-16}$
		Monkey J	0.21	$p = 1.8 \times 10^{-2}$
		Monkey Q	0.42	$p < 2.2 \times 10^{-16}$
Critical bandwidth test	Hall & York, 2001	Both monkeys	26.27	$p < 2.2 \times 10^{-16}$
		Monkey J	24.15	$p = 2.6 \times 10^{-2}$
		Monkey Q	27.97	$p < 2.2 \times 10^{-16}$
Excess mass test	Ameijeiras-Alonso et al., 2019	Both monkeys	0.07	$p < 2.2 \times 10^{-16}$
		Monkey J	0.07	$p = 3 \times 10^{-2}$
		Monkey Q	0.09	$p < 2.2 \times 10^{-16}$

Table S1 | Modality test results for intrinsic neural timescales, related to Fig. 2.

Results of statistical tests assessing the modality of intrinsic neural timescale distributions in the frontal eye field (FEF). The table reports the test statistic and p values for the Hall–York critical bandwidth test, the Fisher–Marron Cramér–von Mises test, and the excess mass test for both monkeys combined $(n = 380)$ and for each monkey separately (Monkey J: $n = 167$; Monkey Q: $n = 205$). Source data are provided as a Source Data file.
