# Supplementary material for: Selective control of prefrontal neural timescales by parietal cortex
Source: Nat Commun. 2026 Mar 9;17:3687. doi: 10.1038/s41467-026-70326-1 (PMC13100148; doi:10.1038/s41467-026-70326-1)
Supplement: Supplementary file 2 — Reporting Summary [file 41467_2026_70326_MOESM2_ESM.pdf]

nature portfolio

Corresponding author(s): Xiaomo Chen

Last updated by author(s): Jan 15, 2026

Reporting Summary

Nature Portfolio wishes to improve the reproducibility of the work that we publish. This form provides structure for consistency and transparency in reporting. For further information on Nature Portfolio policies, see our Editorial Policies and the Editorial Policy Checklist.

Statistics

For all statistical analyses, confirm that the following items are present in the figure legend, table legend, main text, or Methods section.

n/a	Confirmed	
☐	☒	The exact sample size (n) for each experimental group/condition, given as a discrete number and unit of measurement
☐	☒	A statement on whether measurements were taken from distinct samples or whether the same sample was measured repeatedly
☐	☒	The statistical test(s) used AND whether they are one- or two-sided<br>*Only common tests should be described solely by name; describe more complex techniques in the Methods section.*
☐	☒	A description of all covariates tested
☐	☒	A description of any assumptions or corrections, such as tests of normality and adjustment for multiple comparisons
☐	☒	A full description of the statistical parameters including central tendency (e.g. means) or other basic estimates (e.g. regression coefficient) AND variation (e.g. standard deviation) or associated estimates of uncertainty (e.g. confidence intervals)
☐	☒	For null hypothesis testing, the test statistic (e.g. F, t, r) with confidence intervals, effect sizes, degrees of freedom and P value noted<br>*Give P values as exact values whenever suitable.*
☒	☐	For Bayesian analysis, information on the choice of priors and Markov chain Monte Carlo settings
☒	☐	For hierarchical and complex designs, identification of the appropriate level for tests and full reporting of outcomes
☐	☒	Estimates of effect sizes (e.g. Cohen's d, Pearson's r), indicating how they were calculated

Our web collection on statistics for biologists contains articles on many of the points above.

Software and code

Policy information about availability of computer code

Data collection	Experiments were conducted in two adult rhesus macaque (Macaca mulatta) obtained from the Davis Primate Center. Electrophysiological signals were recorded using an OmniPlex Neural Data Acquisition System (Plexon, Inc.), and eye position was monitored with an EyeLink 1000 Plus eye tracker (SR Research). All data were digitized and streamed to disk in real time for subsequent processing. Further details regarding data collection and experimental procedures are described in the Methods section of the manuscript.
Data analysis	Spike sorting was performed with Offline Sorter (version 3.0; Plexon). Subsequent analyses in this study were carried out using R (v4.4.1) in RStudio (v2024.4.2.764).<br><br>The analysis code used to perform all statistical analyses and generate all figures is publicly available in a Code Ocean capsule: https://codeocean.com/capsule/0814945/tree/v1. The capsule includes a README file with instructions to reproduce the analyses and figures.

For manuscripts utilizing custom algorithms or software that are central to the research but not yet described in published literature, software must be made available to editors and reviewers. We strongly encourage code deposition in a community repository (e.g. GitHub). See the Nature Portfolio guidelines for submitting code & software for further information.

Data

Policy information about availability of data

All manuscripts must include a data availability statement. This statement should provide the following information, where applicable:

- Accession codes, unique identifiers, or web links for publicly available datasets
- A description of any restrictions on data availability
- For clinical datasets or third party data, please ensure that the statement adheres to our policy

The data generated in this study have been deposited in the Figshare database (DOI: 10.6084/m9.figshare.28971866). Source data are provided with this paper.

Research involving human participants, their data, or biological material

Policy information about studies with human participants or human data. See also policy information about sex, gender (identity/presentation), and sexual orientation and race, ethnicity and racism.

Reporting on sex and gender	N/A
Reporting on race, ethnicity, or other socially relevant groupings	N/A
Population characteristics	N/A
Recruitment	N/A
Ethics oversight	N/A

Note that full information on the approval of the study protocol must also be provided in the manuscript.

Field-specific reporting

Please select the one below that is the best fit for your research. If you are not sure, read the appropriate sections before making your selection.

☒ Life sciences ☐ Behavioural & social sciences ☐ Ecological, evolutionary & environmental sciences

For a reference copy of the document with all sections, see nature.com/documents/nr-reporting-summary-flat.pdf

Life sciences study design

All studies must disclose on these points even when the disclosure is negative.

Sample size	Two adult male rhesus macaques (Macaca mulatta; monkeys Q and J) participated in this study, in keeping with standard practice for non-human primate neurophysiology. Across all experimental sessions, we recorded activity from 400 neural units in the frontal eye field (FEF).
Data exclusions	We excluded neurons whose single-exponential autocorrelation fit had a pseudo-$R^2 < 0.3$, resulting in a final dataset of 380 units under control conditions and 372 units during parietal inactivation. No other data were excluded.
Replication	Key findings (e.g., selective changes in intrinsic neural timescales) were observed consistently across both monkeys, indicating reproducibility of results. We additionally assessed the stability of intrinsic timescale estimates using a split-half analysis, computing timescales separately from the first and second halves of each recording session, which showed strong agreement between halves.
Randomization	Stimulus location (from a 6×4 grid) and color (red or green) were randomized on each trial. Parietal inactivation was performed in dedicated blocks interspersed with control blocks, and in some sessions the order of blocks (control, inactivation) was varied (control–inactivation vs. control–inactivation–control–inactivation) to reduce potential order effects.
Blinding	Because the experimenter had to implement and monitor cooling during the inactivation blocks, blinding to condition (inactivation vs. control) was not possible during data collection. Data analysis was not blinded.

Reporting for specific materials, systems and methods

We require information from authors about some types of materials, experimental systems and methods used in many studies. Here, indicate whether each material, system or method listed is relevant to your study. If you are not sure if a list item applies to your research, read the appropriate section before selecting a response.

Materials & experimental systems

n/a	Involved in the study
☒	☐ Antibodies
☒	☐ Eukaryotic cell lines
☒	☐ Palaeontology and archaeology
☐	☒ Animals and other organisms
☒	☐ Clinical data
☒	☐ Dual use research of concern
☒	☐ Plants

Methods

n/a	Involved in the study
☒	☐ ChIP-seq
☒	☐ Flow cytometry
☒	☐ MRI-based neuroimaging

Animals and other research organisms

Policy information about studies involving animals; ARRIVE guidelines recommended for reporting animal research, and Sex and Gender in Research

Laboratory animals	Two healthy adult male rhesus macaques (Macaca mulatta; 16 kg and 17 kg) obtained from the California National Primate Research Center (UC Davis) were used in this study.
Wild animals	No wild animals were used.
Reporting on sex	Both animals were male; sex-based analyses were not performed.
Field-collected samples	No field-collected samples were used.
Ethics oversight	All experimental procedures conformed to the guidelines of the National Institutes of Health Guide for the Care and Use of Laboratory Animals, the Society for Neuroscience Guidelines and Policies, and the Stanford University Animal Care and Use Committee. The study protocol was approved by the Stanford University Institutional Animal Care and Use Committee.

Note that full information on the approval of the study protocol must also be provided in the manuscript.

Plants

Seed stocks	N/A
Novel plant genotypes	N/A
Authentication	N/A
